# From Brussels to Mashhad, Professor Roch (Abdullah) Boulvin (1912‒1969), Accelerator of Progression of Modern Surgery in Northeastern Iran

**DOI:** 10.34172/aim.31067

**Published:** 2024-11-01

**Authors:** Ali Emadzadeh, Shirin Taraz Jamshidi, Maryam Emadzadeh, Seyedeh Maryam Mousavi

**Affiliations:** ^1^Department of Internal Medicine, Faculty of Medicine, Mashhad Medical Sciences, Islamic Azad University, Mashhad, Iran; ^2^Department of Pathology, Faculty of Medicine, Mashhad University of Medical Sciences, Mashhad, Iran; ^3^Clinical Research Development Unit, Ghaem Hospital, Mashhad University of Medical Sciences, Mashhad, Iran; ^4^Department of English Language, Faculty of Ethics and Foreign Languages, Islamic Azad University, Mashhad Branch, Mashhad, Iran

 نام نیکو گر بماند ز آدمی ، به کز او ماند سرای زرنگار

 “Passing a good reputation down to others, is much better than leaving wealth as valuable as a golden palace.”

 Saadi Shīrāzī, Iranian Poet, 13th Century

 Born in 1912 in French Guiana, in South America, Roch was the first child of “Georges Jules Adolphe Boulvin” and “Léna Irma Virginie Labenne”, a Belgian couple who lived in the mentioned colony of France at that time due to their jobs.^1,2^ They returned to their home country, Belgium, a few years later. Roch went to medical school in Brussels, Belgium in 1936. and obtained his medical degree in May 1940. He became a resident in the Department of Surgery as the assistant of Professor Duheron. Then, he became a member of the Institute of Pharmacology at Brussels.^2^

 In 1955, he was invited to Mashhad,^3^ the largest and most populous Iranian city after Tehran, located in the northeast. Mashhad University and its medical school were established in 1949, but in those days, this university suffered from a drastic shortage of academic medical staff in some fields, including general surgery.

 Imam Reza Hospital (formerly called Shah Reza Hospital) was the main university hospital in Mashhad (and nowadays is still one of the two most important university hospitals in Mashhad). This hospital belonged to Astan Quds Razavi (A.Q.R.), an organization that manages consecrated properties belonging to Imam Reza Holy Shrine, the 8th religious leader of Shia Muslims. At that time, A.Q.R. managed this hospital in collaboration with the University of Mashhad. This organization invited experts from Western European countries in the 1950s and 1960s to enrich the medical staff of Imam Reza Hospital.^4^

**Figure 1:**
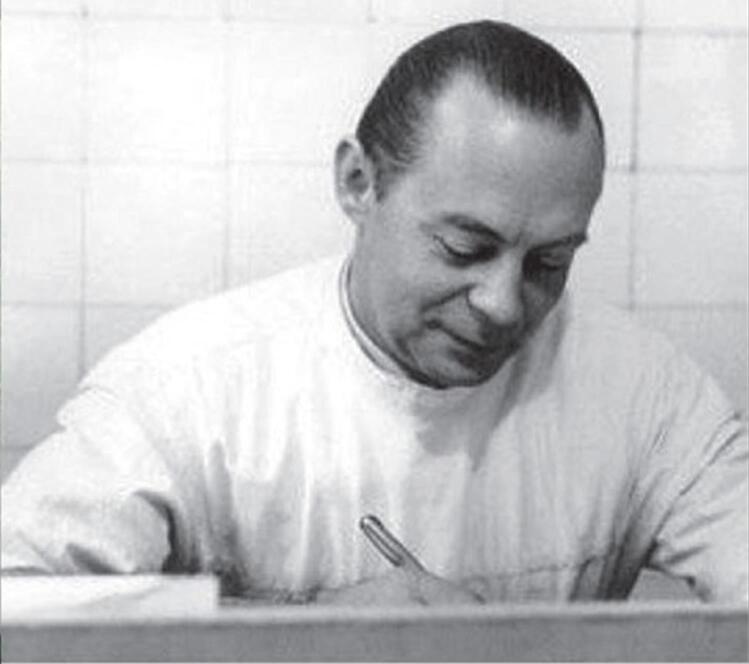
Dr. Roch Boulvin at Operating Room of Imam Reza Hospital, Mashhad, Iran, 1950s – 1960s. Adapted from: Azari Khakestar Gh. “Dar Jostojouy-e Mosalmani” (In Search of Being a Muslim: Dr. Boulvin`s Life and His Perofessional Services) [in Persian]. Bein Al-Nahrain Publication, Mashhad, 2017.^2^

**Figure 2:**
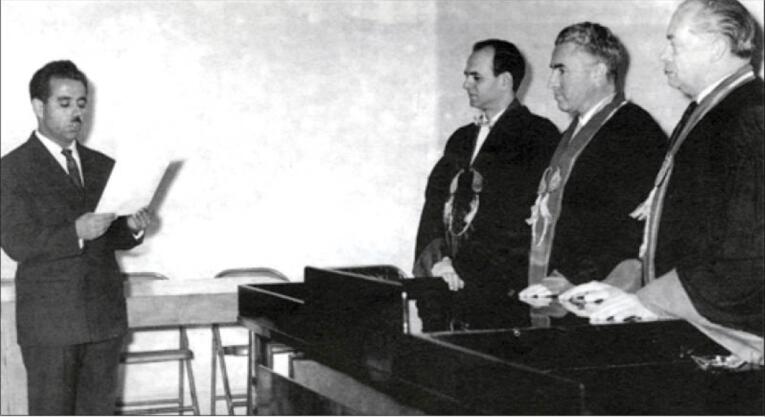
A Dissertation Defense Meeting in front of Mashhad University Jury Board: from right to left: Dr. Roch Boulvin, Dr. Mohammad Shahinfar, Dr. Fakhr Al-din Mahallati, Mashhad, 1962. *Adapted from: Azari Khakestar Gh. “Dar Jostojouy -e Mosalmani ” (In Search of Being a Muslim: Dr. Boulvin`s Life and His Perofessional Services) [in Persian]. Bein Al- Nahrain Publication, Mashhad, 2017.*^2^

**Figure 3:**
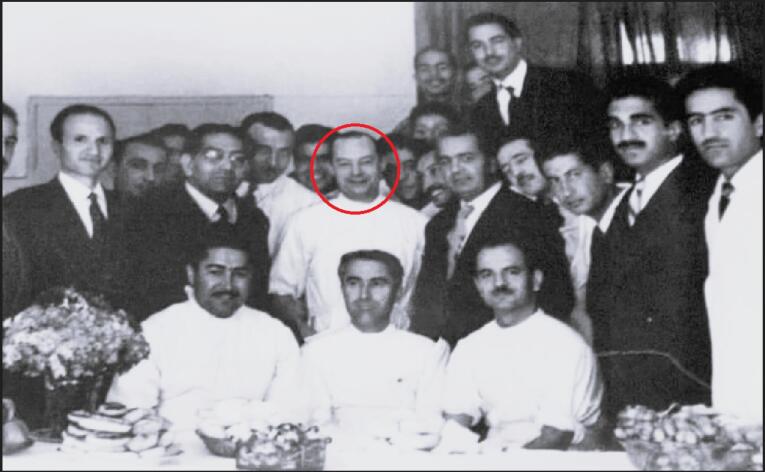
Dr. Roch Boulvin (shown at the center of the picture) and his colleagues, Imam Reza Hospital, Mashhad, 1960s. Adapted from: Azari Khakestar Gh. “Dar Jostojouy-e Mosalmani” (In Search of Being a Muslim: Dr. Boulvin`s Life and His Perofessional Services) [in Persian]. Bein Al-Nahrain Publication, Mashhad, 2017.^2^

 In response to A.Q.R.’s invitation, Dr. Roch Boulvin came to Iran and arrived in Mashhad on February 6, 1955.^2^ He became the head of the Department of Surgery at Imam Reza Hospital from February 12, 1955, until his death on August 5, 1969 (Figures 1-3).^2^

 As the head of the department of surgery, he noticed some major defects and tried to correct them. As a result of his efforts, on the morning of August 23, 1964, the modern operating theater of Imam Reza Hospital (including five separate operation rooms) was opened in the presence of local authorities of the province. He reorganized the rooms with modern equipment, such as central X-ray imaging devices. In addition, Dr. Boulvin started renewing the human resources in surgery ward of the hospital. He rearranged the medical staff and nursing teams and implemented regular night shifts for general practitioners and surgery residents. Thus, many European-experienced nurses like Mlle. M. Mary Tayar, who became one of the best colleagues of Dr. Boulvin, were invited to the Department of Surgery. Boulvin also established a blood bank to revolutionize patient management in the hospital.^2^

 Boulvin cooperated with the department of surgery at the University of Brussels. He compiled numerous articles and published them in high-ranking medical journals worldwide, mostly French and Belgian journals.^2,3^ Most of these articles were the results of his own experiences with his patients. A variety of research fields are evident in his articles, ranging from gastrointestinal tuberculosis and Potts Disease to thyroid cancer and other diseases in the field of general surgery.

 Some of Professor Boulvin’s published papers in French and Belgian medical journals (in the 1950s and 1960s) are as follows:

1. Boulvin R. [First impressions on the direct approach of Pott’s disease in Iran]. Acta orthopaedica Belgica. 1957;23(5-6):334-50.^5^
2. Boulvin R. [Hepatic hydatidosis in Iran]. Annales de chirurgie. 1962;16:1259-68.^6^
3. Boulvin R. [Duodenal Ulcer in Iran]. Acta gastro-enterologica Belgica. 1964;27:727-49.^7^
4. Boulvin R. [Peritonitis caused by typhic perforation in Iran]. Annales de chirurgie. 1965;19(11):785-9.^8^
5. Boulvin R. [Jaundice associated with hepatic hydatidosis in Iran]. Memoires Academie de chirurgie (France). 1967;93(15):484-6.^9^
6. Boulvin R, Chahinfar M. [Thyroid cancer in Iran]. Revue medicale du Moyen-Orient. 1965;22(2):280-8.^10^

He also developed some surgical procedures, such as splenoportography, using his own unique approach using direct transparietal injection of dye into the splenic bulb.^11,12^ At that time, the American Gastroenterological Association recognized him as one of the pioneers of this method in the world.^13^

**Figure 4:**
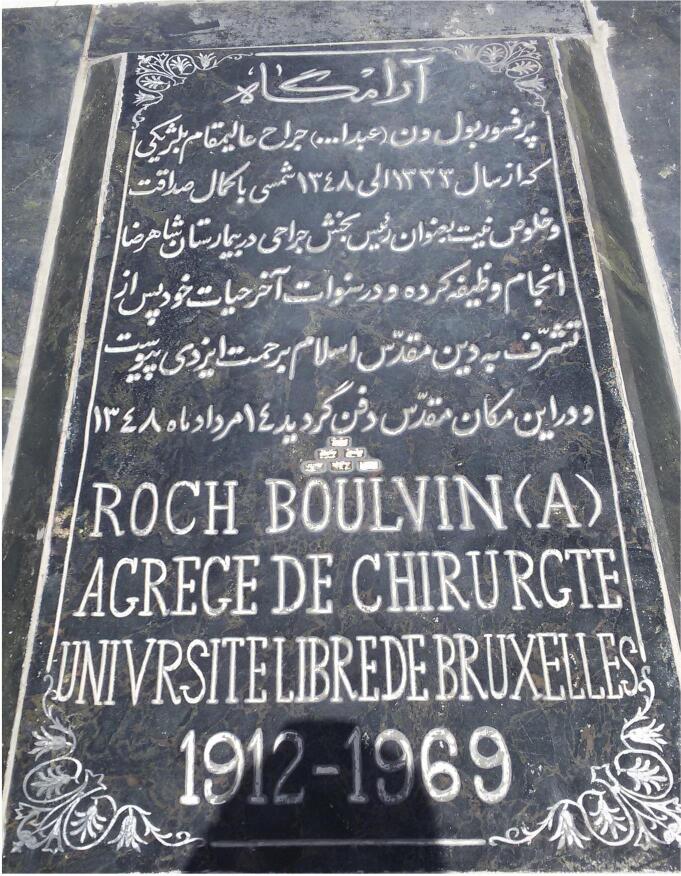
Tombstone of Dr. Abdullah (Roch) Boulvin in Khajeh Rabi’ cemetery, Mashhad, Photo taken by Ali Emadzadeh (first author), June 2015.

 Dr. Roch Boulvin lived in Mashhad until his death for about 14 years. He performed hundreds of surgeries, treated thousands of patients, and published many research papers in medical journals. With his general discipline, re-arrangement of surgical equipment and human resources, kindness with patients, and great attention to medical research studies, he made an unforgettable impression on the academic community and general population of Mashhad. He converted to Shia Islam in the last days of his life and changed his name from Roch to Abdullah.^1-3^ He passed away on Tuesday morning, August 5, 1969. His funeral ceremony was held gloriously with a large group of people, including his patients, colleagues, medical and academic staff, along with the mayor and other local authorities of Mashhad.^2^ His body was buried as a Muslim in Khajeh Rabi cemetery in the northern area of Mashhad^3,4^ (Figure 4).

 After Abdullah’s death, his wife, Mrs. Adrienne Marie Jacqueline, returned to Belgium. Adrienne taught French Language at the Faculty of Humanities of Mashhad during their residence in Iran.^2^ She passed away in Brussels at 95 on February 18, 2017.^1,14^ The couple had two sons, Michel and Claude Georges. We could not find any information about Claude, but it seems that Michel, born in 1943, is alive at the time of writing this manuscript.^1,14^

**Figure 5:**
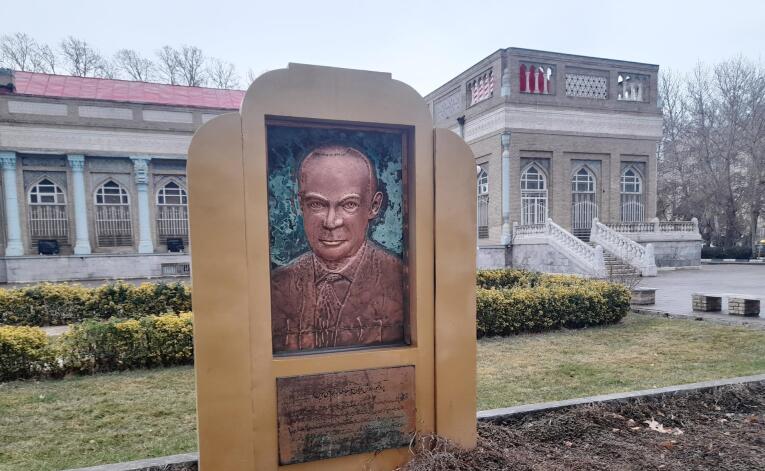
The statue of Dr. Abdullah (Roch) Boulvin at the courtyard of Imam Reza Hospital, Mashhad. Photo taken by Shirin Taraz Jamshidi (second author), March 2024.

 Despite more than half a century after Dr. Abdullah Boulvin’s death, the general population and the old medical staff of Mashhad still remember him well because of his endless fame. Due to his valuable services and excellent administrative and medical reformation, Dr. Boulvin is still alive in the hearts of the people of Mashhad (Figure 5). He has illustrated a prominent instance of the poem of Sa’di, an Iranian poet of the 13th century which was quoted at the beginning of this paper:

 “Passing a good reputation down to others, is much better than leaving wealth as valuable as a golden palace.”
